# Compact wideband plasmonic filter with flat-top transmission response based on corrugated metal-insulator-metal ring resonator

**DOI:** 10.1038/s41598-017-14708-y

**Published:** 2017-10-27

**Authors:** Liu Yang, Yong Jin Zhou, Chao Zhang, Qian Xun Xiao

**Affiliations:** Key Laboratory of Specialty Fiber Optics and Optical Access Networks, Shanghai University, Shanghai, 200444 China

## Abstract

We demonstrate a novel route to control the filtering of spoof localized surface plasmons (LSPs) on the corrugated metal-insulator-metal (MIM) ring resonator. The spoof LSPs resonance modes can be effectively tuned to achieve broad passband (covering the quadrupole mode and the hexapole mode) by selecting proper lengths in the input and output coupling area. The mutual coupling between the input and output lines produces the flat-top transmission response and sharp out-of-band rejection. Compared with the wideband bandpass filters based on spoof plasmonic waveguides, the proposed filter is ultra-compact and only 0.35λ*0.35*λ*. In order to further improve the property of the bandpass plasmonic filter, all the out-of-band frequencies (the dipole mode and the octopole mode) have been rejected by introducing a shunt stepped-impedance resonator and double C-shaped rings on the back of the substrate of the filter. Simulated results are confirmed via experiment, showing good rejection and wideband filtering performance with low insertion loss, flat-top transmission response and sharp out-of-band suppression. The proposed filter can find more applications in the highly integrated plasmonic circuits and systems in both terahertz and microwave regimes.

## Introduction

Surface plasmons (SPs) have attracted enormous research attention in recent years, due to their capabilities of breaking the classical diffraction limit and manipulating light at the sub-wavelength scale^1^. SPs exist either as propagating surface plasmon polaritons (SPPs) on the extended interface between metal and dielectric or localized surface plasmons (LSPs) on finite metal particles^2^. The study of generating and manipulating SPs has been expanded from the optical regime to microwave and terahertz (THz) regime, thanks to the pioneering work from Pendry *et al*.^3^. These surface modes on the structured metal surface are widely termed as designer or spoof SPs. These spoof plasmonic structures possess not only similar capacity of field confinement and non-diffraction limit as that of optical SPs, but also the flexible controllability by tailoring the geometrical parameters on patterns due to their millimeter scale size. While most of these studies focused on propagating spoof SPPs^4–6^, recently, spoof LSPs have been demonstrated using spoof plasmonic resonators^7–10^.

Spoof SPPs waveguides are often used to design wideband bandpass filter^11–15^. Among the various plasmonic waveguides, the metal-insulator-metal (MIM) waveguides allow the highly confined SPPs modes to propagate in a sharp bend with low additional transmission loss and a simple fabrication technique^16^. Spoof plasmonic analogue of MIM waveguides was then proposed^17^ and ultra-wideband bandpass filters based on spoof MIM waveguides have been studied at microwave frequencies^18–20^. However, efficient conversions between spoof SPPs and guided waves are necessary for feeding energies into and extracting signals from functional plasmonic devices through transmission lines. Hence, the length of these wideband filters in the propagation direction is typically several wavelengths. Although LSPs resonators are compact, they are usually used to implement narrowband filters with asymmetrical teeth-shaped structure^21^, MIM nanodisk cavity^22^, corrugated ring resonator^23^, etc. Besides, by controlling the couplings between the spoof LSPs particles and the spoof SPPs waveguides^24,25^, narrowband rejection filters have been proposed. To broaden the operating band, different structures such as a symmetrical multiple-teeth-shaped structure^26^, a metal bar loaded into the stub^27^, a graded plasmonic resonator chain^28^, an ultra-thin periodic corrugated metallic strip with defect units^29^ and so on, have been investigated. However, such methods will not only increase the filter length, but also bring in additional transmission loss. Moreover, most of plasmonic filters mentioned above have a Lorentzian-shaped transmission response, not the flat-top transmission response which is required for many communication systems. Squared ring resonators^30^ and cascaded rectangular ring resonators^31^ have been designed as bandpass filters with flat-top transmission response. To the best of our knowledge, there is no reported work on plasmonic wideband bandpass filter with flat-top transmission response based on spoof LSPs resonators.

The corrugated MIM ring resonator has been fully investigated in Ref.^32^. In this paper, an ultra-compact and wideband bandpass plasmonic filter based on the corrugated MIM ring resonator has been numerically and experimentally demonstrated. Different from almost all of the above filters, where their resonance wavelengths are often changed by modifying the geometrical parameters of the resonators, a new adjusting mechanism is applied to broaden the transmission band covering the quadrupole mode and the hexapole mode. It has been shown that the spoof LSPs resonance modes can be effectively tuned by selecting proper lengths in the input and output coupling area. By introducing the mutual coupling between the input and output lines, flat-top transmission and sharper out-of-band rejection can be achieved. The whole wideband bandpass filter is only 0.35λ*0.35*λ* and ultra-compact, compared with the previously mentioned wideband bandpass filters based on spoof SPPs waveguides. In order to further improve the property of the bandpass filter, some other methods are used to reject all the frequencies outside the interested passband. By introducing a shunt stepped-impedance resonator (SIR) and double C-shaped rings on the back of the substrate of the filter, the rejections of the dipole mode and the octopole mode have been implemented. Hence, the spoof plasmonic filter exhibits several attractive features such as ultra-compact size, wide bandwidth, low insertion loss, flat-top transmission response and sharp out-of-band suppression. The measured results agree well with the simulated results. The structures can find more applications in the highly integrated plasmonic circuits and systems at microwave and THz frequencies.

## Results and Discussion

### Design of wideband filtering of spoof LSPs

**Figure 1:**
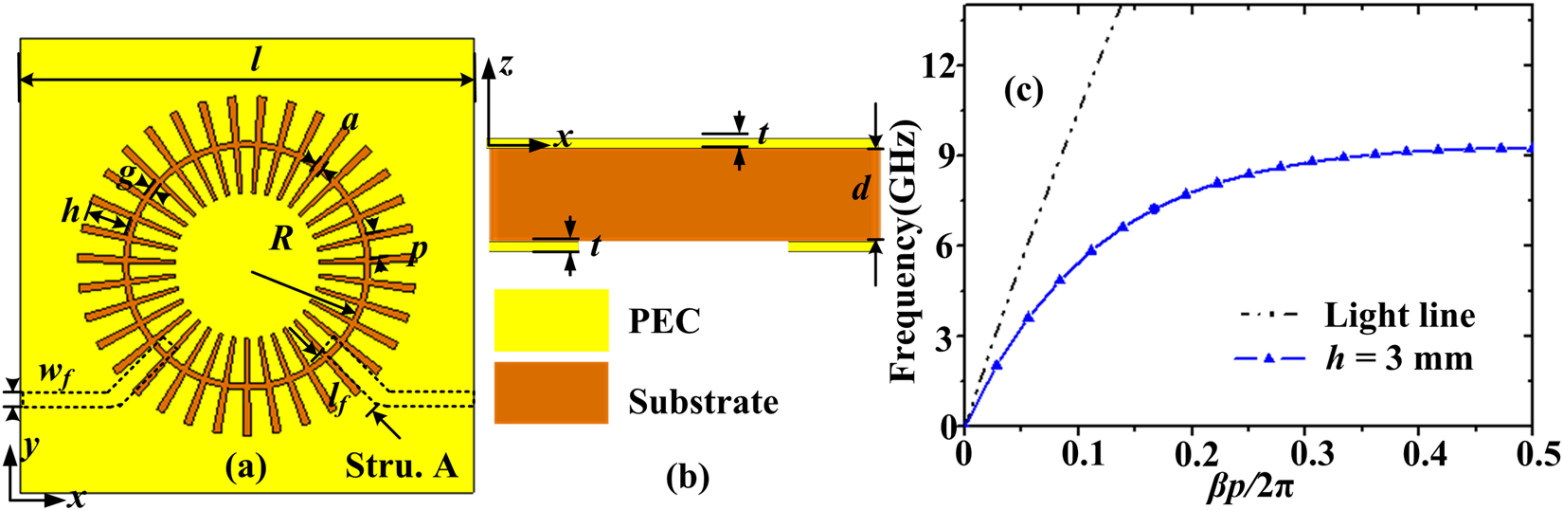
(**a**) The top view and (**b**) the side view of the schematic diagram of the narrowband bandpass filter (structure A) composed of grooved MIM ring resonator and the input and output microstrip lines, where the parameters *l*, *h* and *g* of the MIM ring are 30 mm, 3 mm and 0.4 mm, the metal thickness *t* and the substrate thickness *d* are 18 μm and 0.635 mm, and *w*_*f*_ and *l*_*f*_ of the microstrip line are 0.64 mm and 5 mm, respectively. (**c**) Dispersion curve of spoof SPPs on the corrugated MIM strips when the groove depth *h* is 3 mm.

The corrugated MIM ring resonator is composed of two closed corrugated metal strips printed on the substrate Rogers RO3010 whose thickness *d* is 0.635 mm, as illustrated in Fig. 1(a) and (b). The side length *l* of the structure is 30 mm. The metal thickness *t* is 18 μm. The number *N* of the grooves is 35 and the groove depth *h* is chosen as 3 mm. Both the groove period and groove width of the corrugated MIM ring resonator (on the plane *z* = 0) are non-uniform. The groove period is determined by 2*πR*/*N* and the groove width is 2πR*2.8/360, where *R* is the local radius of the ring. For simplicity, the insulator of the MIM ring is set to air and its inner radius is set as 7.8 mm. The width *g* of the air is 0.4 mm. The dispersion characteristics have been calculated and shown in Fig. 1(c), where the mean groove width and groove period are used and they are 0.40 mm and 1.47 mm, respectively. It has been verified that the spoof LSPs are actually standing waves and they satisfy the relation *L* ≈ *n*∙*λ*_*g*_, where *L* is the circumference of the air ring, *λ*_*g*_ is the guided wavelength on the corrugated MIM resonator and *n* is the mode number^32^.

**Figure 2:**
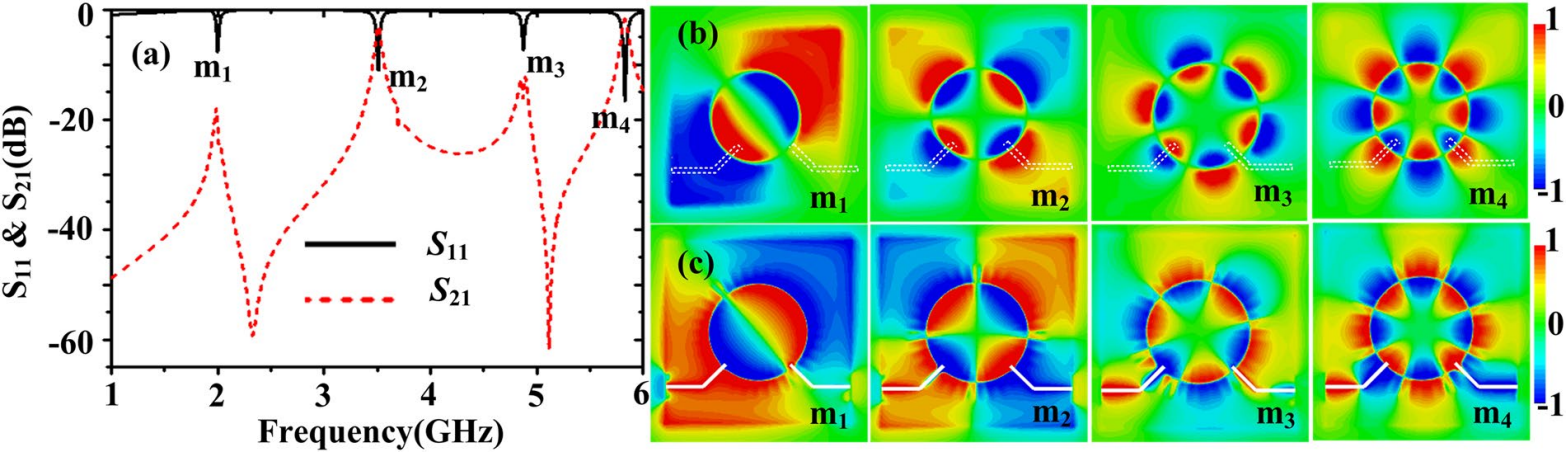
(**a**) Reflection and transmission coefficients of the spoof plamonic narrowband bandpass filter (structure A). 2D *E*_*z*_-field distributions on the plane (**b**) *z* = 1 mm and (**c**) *z* = −0.635 mm of the narrowband bandpass filter (structure A).

In ref.^32^, it has been shown that spoof LSPs modes in the corrugated MIM ring can be efficiently excited by use of a microstrip line and the exciting efficiency depends on the matching degree between the magnetic field of the exciting source and that in the corrugated MIM ring. Here, by adding an output microstrip line which is perpendicular to the input microstrip feed line (see the dashed lines in Fig. 1(a)), a narrowband bandpass filter denoted as structure A can be implemented. The width *w*_*f*_ and length *l*_*f*_ of the 50 Ω microstrip line are 0.64 mm and 5 mm, respectively. The reflection and transmission coefficients of the filter are obtained and given in Fig. 2(a). The resonance modes are marked as m_1_-m_4_, whose resonance frequencies are 2.0 GHz, 3.51 GHz, 4.87 GHz, and 5.83 GHz, respectively. To understand the operating mechanisms, the two-dimensional (2D) *E*_*z*_-field distributions on the *xoy* plane 1 mm above the structure at these resonant frequencies are illustrated in Fig. 2(b). It can be seen that the dipole (*n* = 1), quadrupole (*n* = 2), hexapole (*n* = 3) and octopole (*n* = 4) modes have been effectively excited. Since the electric fields are very weak at the output coupling area for the dipole mode and the hexapole mode, it’s expected that the two odd modes would not be coupled out. From the transmission coefficients in Fig. 2(a), we can see that the odd order modes are suppressed due to the configuration of the output and input microstrip lines, while the even order modes can be coupled out. However, their transmission bandwidths are very narrow. The full width at half maximum (FWHM) is 10 MHz for m_2_ mode and it is 40 MHz for m_4_ mode. The insert losses are −2.99 dB and −1.8 dB, respectively. Furthermore, the transmission response is Lorentzian-shaped. Figure 2(c) illustrates the 2D *E*_*z*_-field distributions on the plane *z* = −0.635 mm (the bottom of the filter structure) at these resonant frequencies. We can see that the field distributions on the plane *z* = −0.635 mm are the same as those on the plane *z* = 1 mm.

**Figure 3:**
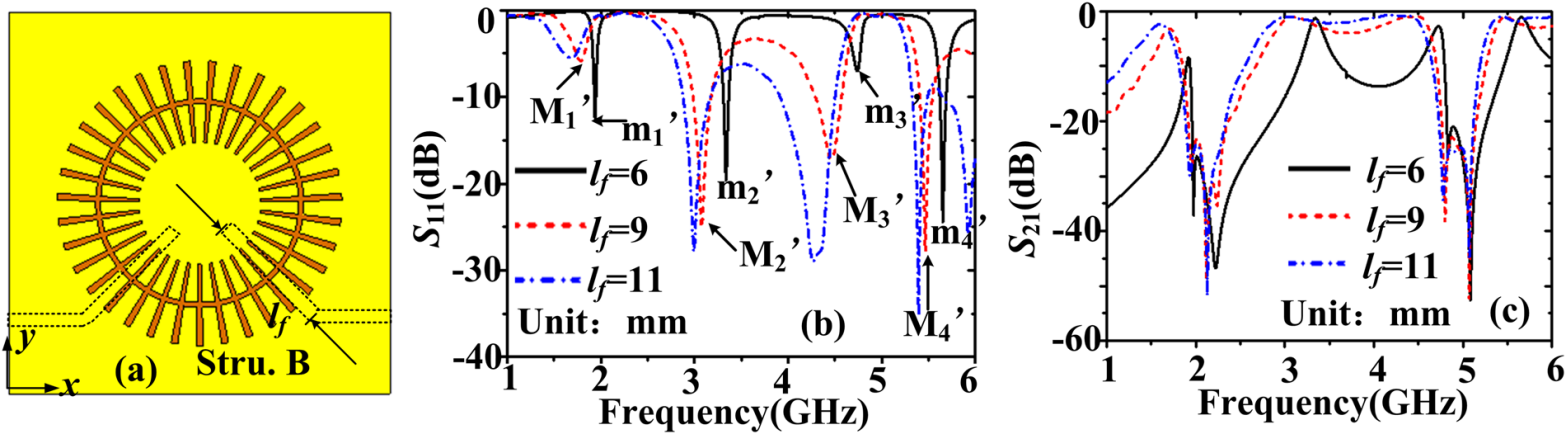
(**a**) The top view of the wideband bandpass filter with *l*_*f*_ = 9 mm (structure B). (**b**) Reflection coefficients and (**c**) transmission coefficients of the wideband bandpass filter when *l*_*f*_ changes from 6 mm to 11 mm.

**Figure 4:**
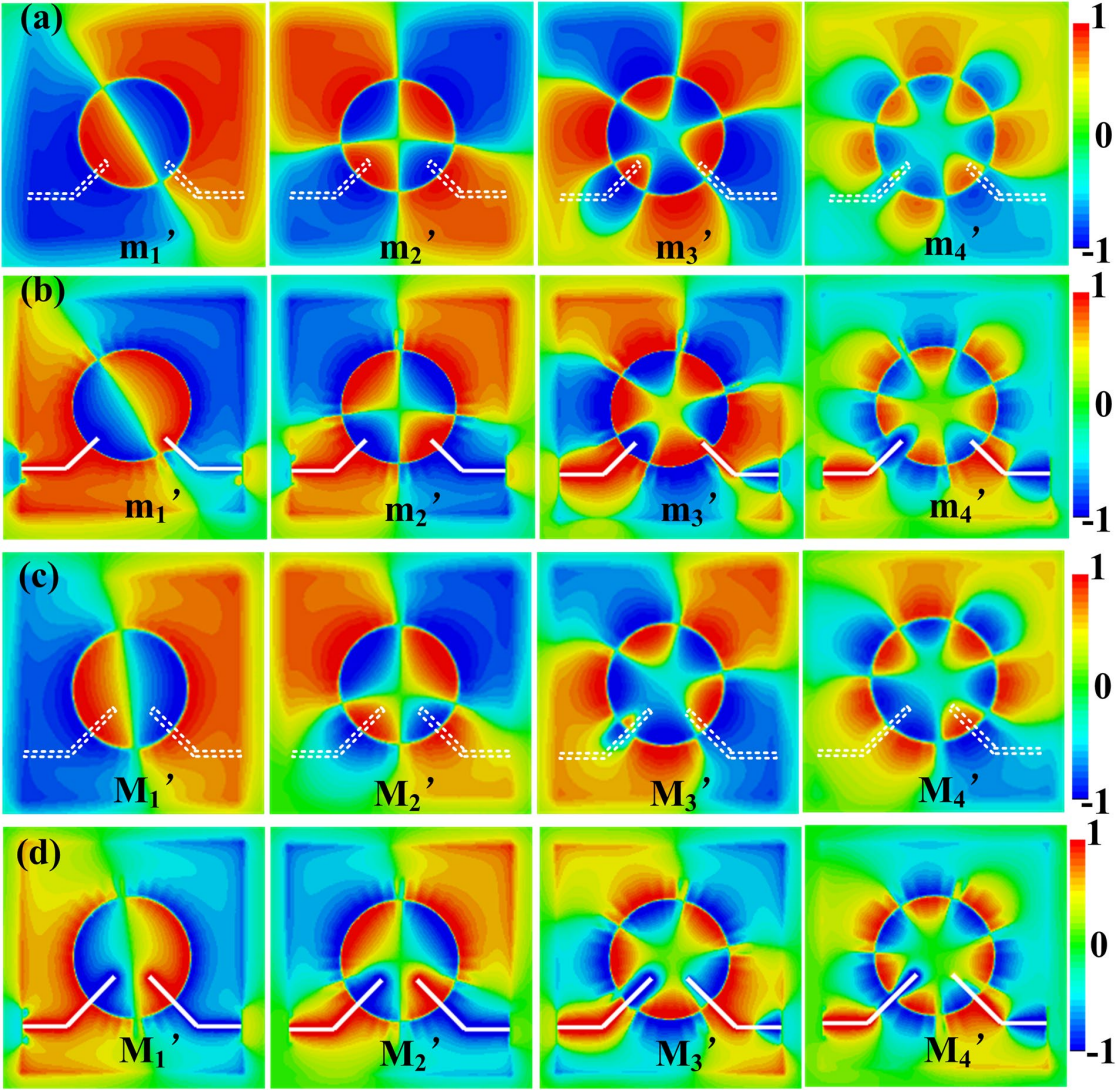
2D *E*_z_-field distributions (**a**) on the plane *z* = 1 mm and (**b**) on the plane *z* = −0.635 mm when *l*_*f*_ = 6mm. 2D *E*_z_-field distributions (**c**) on the plane *z* = 1 mm and (**d**) on the plane *z* = −0.635 mm when *l*_*f*_ = 9 mm.

Next, we will investigate how and why the operating passband can be broadened. When the feeding microstrip is extended approximately one-quarter wavelength beyond the point of crossing, a broadband transition can be achieved^33^. The operating central frequency of the wideband filter is set to 3.5 GHz. Hence, the objective is to efficiently excite the quadrupole (*n* = 2) mode and the hexapole (*n* = 3) mode simultaneously, whose corresponding guided wavelengths are about 24.5 mm and 16.3 mm, respectively. When *l*_*f*_ is increased to 9 mm, the filter is denoted as structure B, shown in Fig. 3(a). When the length *l*_*f*_ of the input and output microstrip lines changes, the simulated quadrupole mode and the hexapole mode in Fig. 3(b). The new resonance modes are marked as m_1_’–m_4_’ and M_1_’–M_4_’ for *l*_*f*_ = 6 mm and *l*_*f*_ = 9 mm. The resonance frequencies are 1.94 GHz, 3.34 GHz, 4.74 GHz, and 5.66 GHz for m_1_’–m_4_’ modes. For M_1_’–M_4_’ modes, they are 1.77 GHz, 3.06 GHz, 4.43 GHz, and 5.41 GHz, respectively. First, we can see that the reflected waves have been decreased below -5 dB in the band between the M_2_’ mode and M_3_’ mode. The transmission spectra are plotted in Fig. 3(c). There exist two narrow bands (corresponding to the quadrupole mode and the hexapole mode) from 2.5 GHz to 5 GHz when *l*_*f*_ = 6 mm. When *l*_*f*_ is increased to 9 mm, we can see that the transmission band expands and its FWHM becomes 1.64 GHz. At this time, the extended length of the input line beyond the point of crossing is about 4.5 mm which is between one-quarter wavelengths of the quadrupole mode and the hexapole mode (4.075 mm–6.125mm). That is, the length of the input microstrip line is set to be approximate a quarter wavelength of the chosen central frequency (3.5 GHz). Hence, a wideband passband covering the excited two resonant modes can be formed. Second, it can be seen that the resonance frequencies red shift when *l*_*f*_ is increased. Because the extended lengths of the input line beyond the point of crossing are still in the range of one-quarter wavelengths of the quadrupole mode and the hexapole mode (4.075 mm–6.125 mm) when *l*_*f*_ is larger than 9 mm and no larger than 11 mm, broad passpand (covering the quadrupole mode and the hexapole mode) can also be achieved. However, The FWHM is almost unchanged when *l*_*f*_ is larger than 9 mm. For example, the FWHM is 1.67 GHz when *l*_*f*_ is 11 mm which is almost the same as that for *l*_*f* = _9 mm. While the input and output microstrip lines are very close when *l*_*f*_ = 11 mm. Hence, the final length *l*_*f*_ is set to 9 mm. To reveal the physical mechanisms, Fig. 4(a) and (b) illustrate the simulated *E*_*z*_-field distributions for the m_1_’–m_4_’ modes on the plane z = 1 mm and *z* = −0.635 mm, respectively. We can see that the electric filed distributions on the plane *z* = 1 mm are almost the same as those on the plane *z* = −0.635 mm except for the m_4_’ mode. From Fig. 4(b), we can see that the input coupling line constitutes one pole of the octopole mode for the m_4_’ mode, while it is not obvious for the m_3_’ mode. Figures 4(c) and (d) illustrate the simulated *E*_*z*_-field distributions for the M_1_’–M_4_’ modes on the plane z = 1 mm and *z* = −0.635 mm, respectively. With further increase of *l*_*f*_, we can see that the electric field distributions on the plane z = 1 mm and *z* = −0.635 mm are different for M_3_’ and M_4_’ modes. From Fig. 4(d), it can be clearly observed that the input coupling line has constituted one pole of the hexapole mode and octopole mode for the M_3_’ and M_4_’ modes, indicating higher coupling efficiency between the input microstrip line and the corrugated MIM ring resonator. Thus less EM waves are reflected, more EM waves could be coupled out, and the insertion loss is decreased when *l*_*f*_ is 9 mm. Furthermore, the effective circumference length (including the input line) is increased and the resonance frequencies red shift. We can conclude that the increasing length of the input and output coupling lines affects the field distributions. Although the transmission band is broad, we can see that it’s still not flat-top and the out-of-band rejection is not sharp.

### Wideband filtering of spoof LSPs with flat-top transmission response and sharp out-of-band rejection

**Figure 5:**
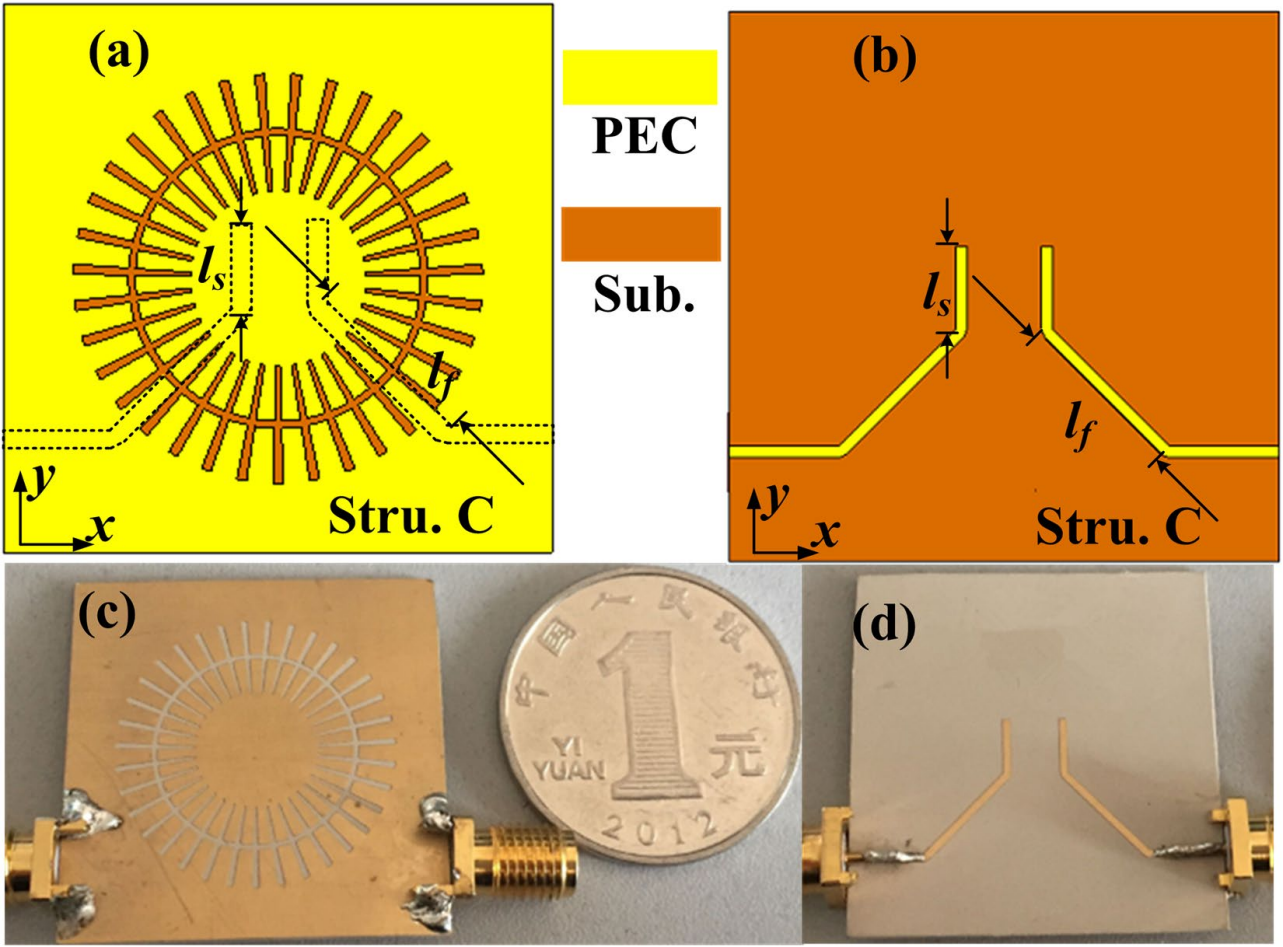
(**a**) The top view and (**b**) the bottom view of the schematic diagram of the wideband bandpass filter with flap-top transmission response (structure C). (**c**) The top view and (**d**) the bottom view of the fabricated wideband bandpass filter with flap-top transmission response.

The lengths of the input and output coupling lines are further increased. The schematic configuration and the sample are shown in Fig. 4 and the filter is denoted as structure C. Figure 5(a) and (b) illustrate the top view and the bottom view of the proposed wideband bandpass filter, where the added stub length is denoted as *l*_*s*_. The fabricated sample is shown in Fig. 5(c) and (d), where the length *l*_*f*_ and *l*_*s*_ of the microstrip line are 9 mm and 4.5 mm, respectively.

**Figure 6:**
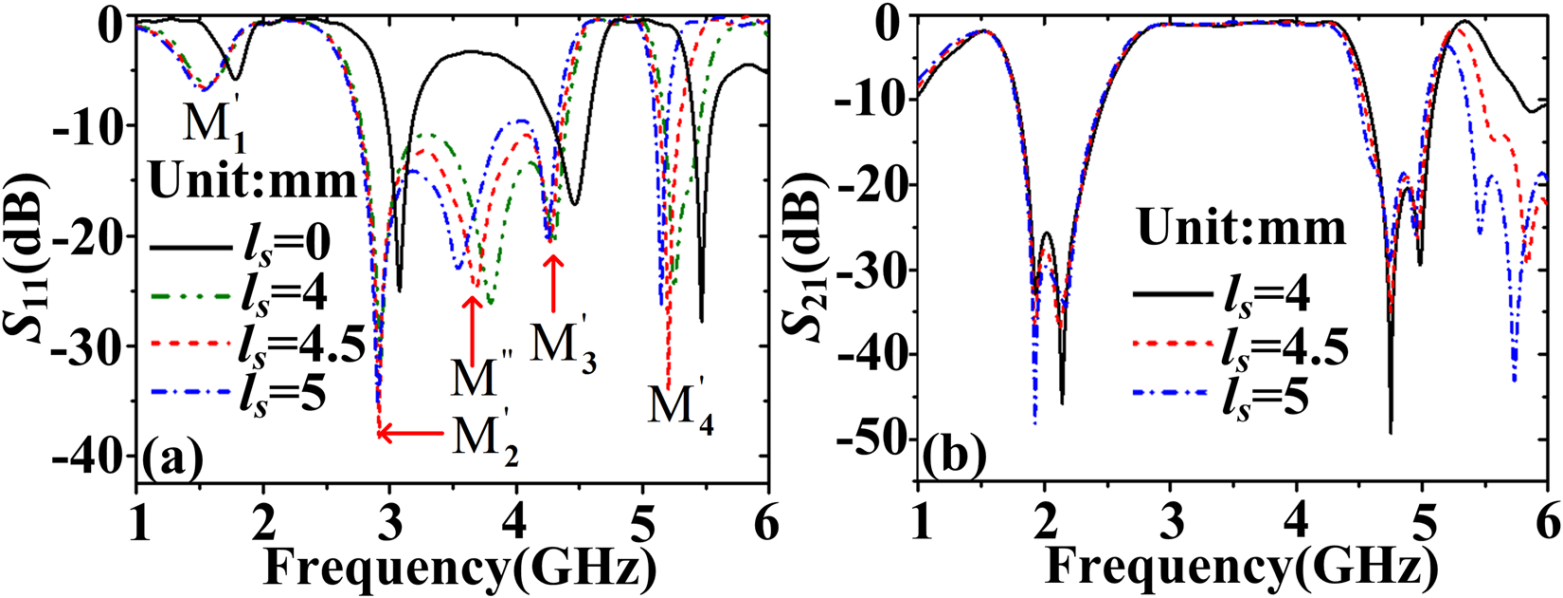
(**a**) Reflection coefficients and (**b**) transmission coefficients of the wideband bandpass filter with flap-top transmission response (structure C) when *l*_*s*_ changes.

The simulated reflection and transmission coefficients corresponding to different length *l*_*s*_ are given in Fig. 6(a) and (b). We can see that there are five resonant modes and their corresponding resonance frequencies are 1.54 GHz, 2.91 GHz, 3.70 GHz, 4.27 GHz, and 5.19 GHz when *l*_*s*_ is 4.5 mm. In addition to the normal resonance modes M_1_’–M_4_’, a new kind of resonance mode marked as M″ appears from Fig. 6(a). For the modes M_1_’–M_4_’, the resonance frequencies red shift compared with the case *l*_*s*_ = 0 from Fig. 6(a). With the increase of the length *l*_*s*_ (from 4 mm to 5 mm), the resonance frequencies of the modes M_1_’–M_4_’ red shift, however, the changes are not obvious. While the redshift of the resonance frequency of the resonance mode M″ is obvious, indicating that this mode may be different from the modes M_1_’–M_4_’. From the results in Fig. 6(b), we can see that the transmission band has become flat-top due to the new mode M″. The transmission band is from 2.65 GHz to 4.43 GHz and the insert loss is only −1 dB. The lower stopband (below -20 dB) is from 1.86 GHz to 2.30 GHz. The upper stopband is from 4.67 GHz to 5 GHz. Moreover, the out-of-band suppression is sharper. Especially, the whole structure is only 0.35λ*0.35*λ*, where *λ* is the wavelength at the center frequency in the passband.

**Figure 7:**
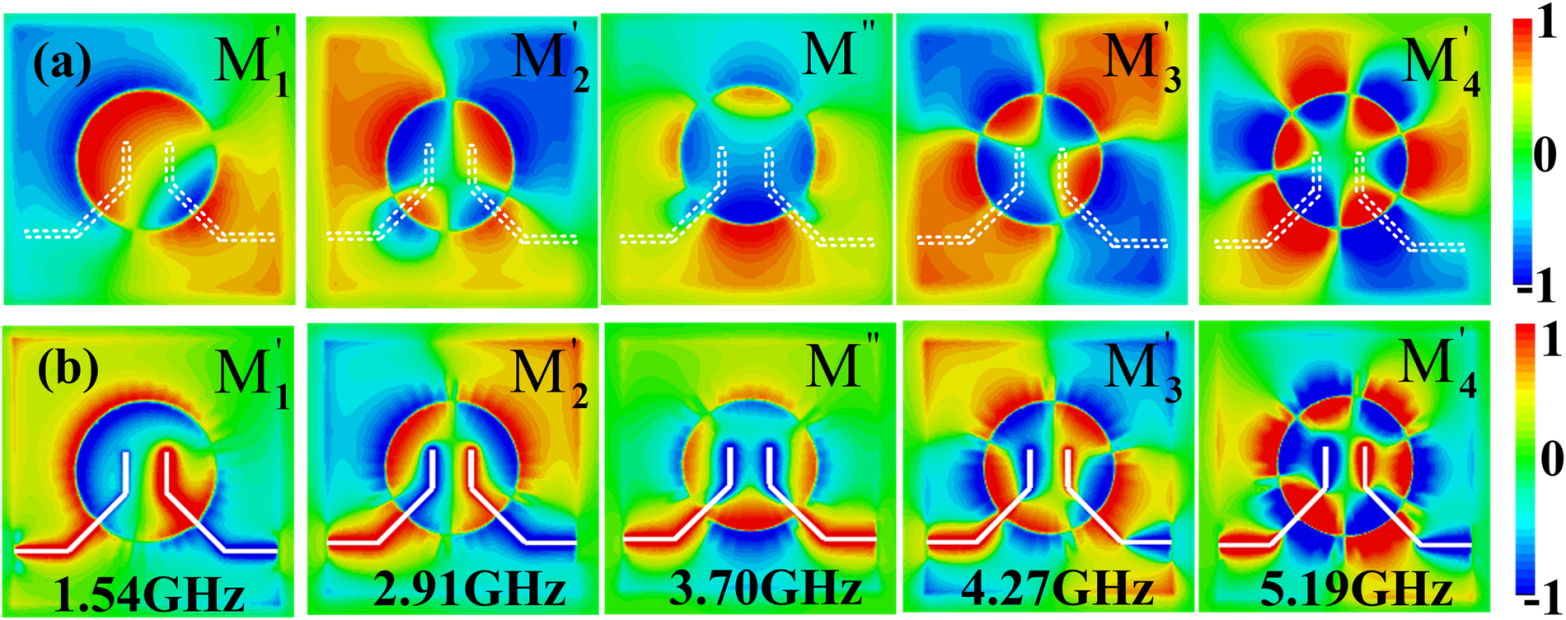
Simulated 2D *E*_*z*_-field distributions (**a**) on the plane *z* = 1 mm and (**b**) on the plane *z* = −0.635 mm of the wideband bandpass filter with flap-top transmission response (structure C) when *l*_*s*_ = 4.5 mm and *l*_*f*_ = 9 mm.

To verify whether M″ is a new resonance mode, the 2D *E*_*z*_-field distributions on the plane *z* = 1 mm and *z* = −0.635 mm at these resonant frequencies have been monitored and illustrated in Fig. 7(a) and (b). From Fig. 7(a), we can see that the M_1_’ and M_2_’ modes are dipole mode and quadrupole mode, while both M″ and M_3_’ mode are quadrupole mode and M_4_’ mode is hexapole mode. However, when we observe the 2D *E*_*z*_-field distributions on the plane *z* = −0.635 mm illustrated in Fig. 7(b), it can be clearly seen that for M_2_’, M_3_’ and M_4_’ modes, the input and output lines have actually become two poles of the quadrupole mode, hexapole mode, and octopole mode and the fields on the lines are out of phase. Different from these resonant modes, the fields for the resonance mode M″ are in phase. Hence, as the length *l*_*s*_ is increased, the effective inductance increases and the resonant frequency of the mode M″ red shifts obviously. Thus we can conclude that the mode M″ stems from the mutual inductive coupling of the input and output microstrip lines. While the modes M_1_’–M_4_’ are from the couplings between the input and output lines and the spoof plasmonic resonator. The changes of the resonance frequencies with the increase of *l*_*s*_ are small.

**Figure 8:**
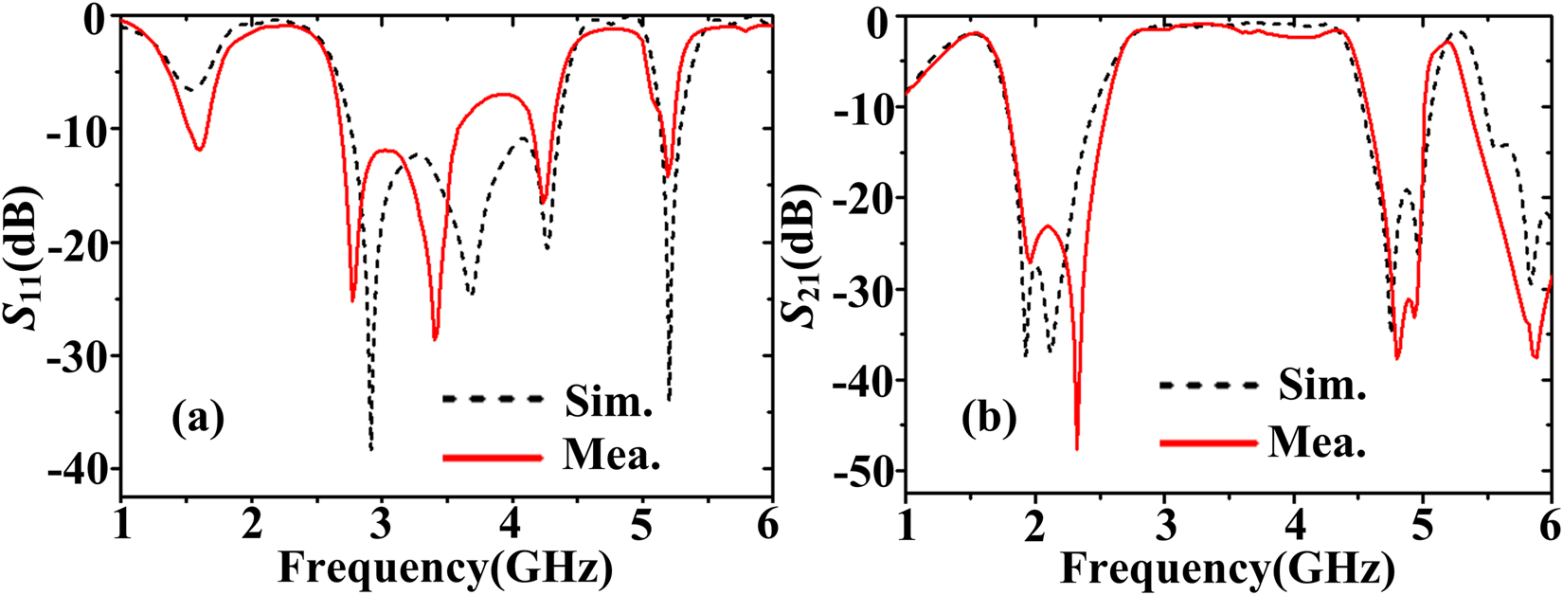
The simulated and measured (**a**) reflection coefficients and (**b**) transmission coefficients of the wideband bandpass filter with flap-top transmission response (structure C) when *l*_*s*_ = 4.5 mm and *l*_*f*_ = 9 mm.

The measured reflection and transmission coefficients are plotted in Fig. 8(a) and (b). The measured resonance frequencies are 1.60 GHz, 2.77 GHz, 3.40 GHz, 4.24 GHz, and 5.19 GHz, respectively, which have good agreements with the simulated results. The slight resonance frequencies deviation could be caused by the dielectric constant difference between the simulation and the sample. The FWHM is from 2.67 GHz to 4.46 GHz and its relative bandwidth is 50.2%. Furthermore, the lower rejection band is from 1.87 GHz to 2.44 GHz. The transmission loss is a little larger than the simulation results at the higher frequencies, which may be due to the higher dielectric loss for the practical substrate. However, the out-of-band rejection in the measurement is sharper.

### Rejection of out-of-band spoof LSPs modes

**Figure 9:**
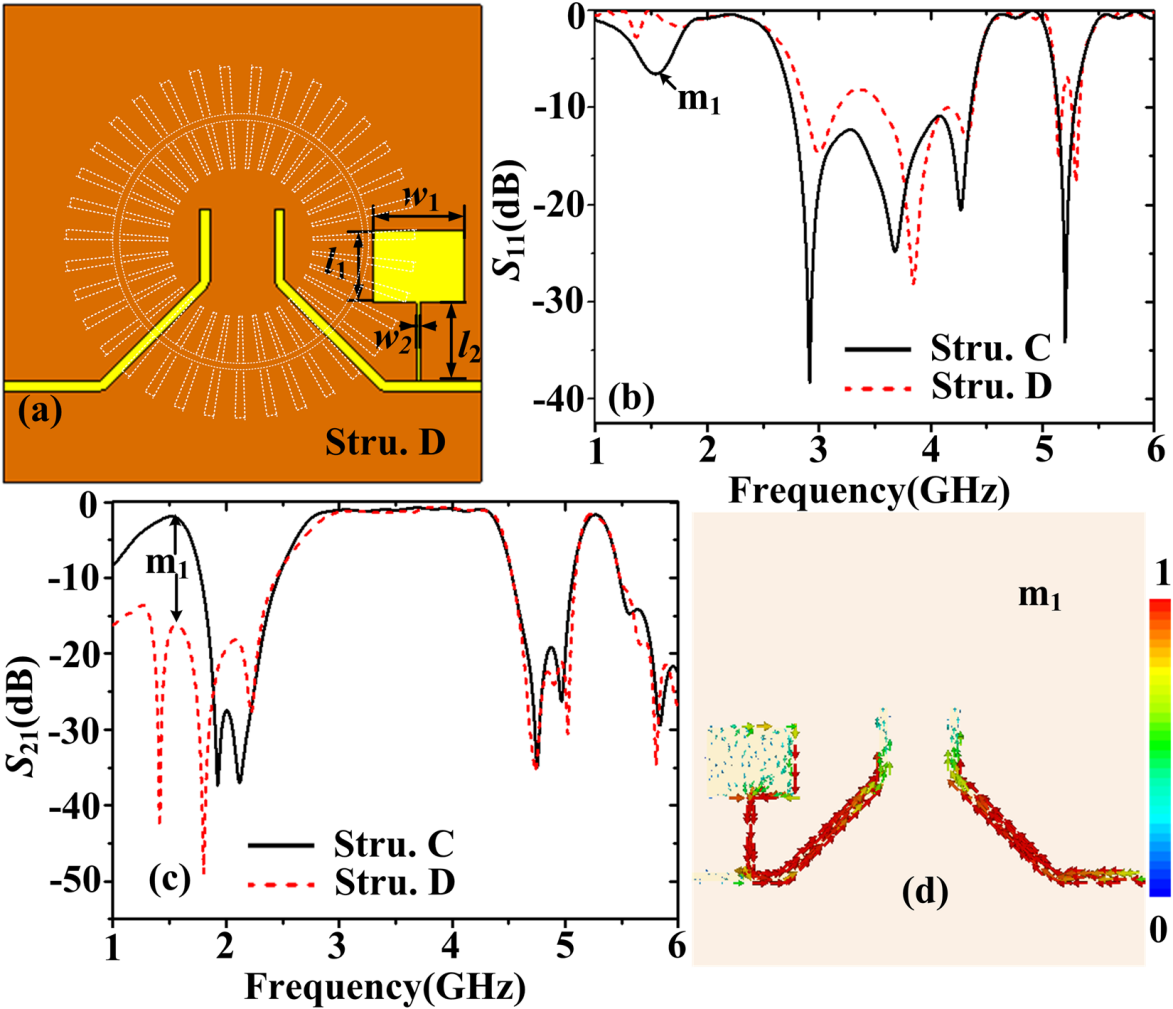
(**a**) The bottom view of the wideband bandpass plasmonic filter with a shunt SIR (structure D), where the parameters *l*_1_, *l*_2_, *w*_1_, and *w*_2_ are 4.5 mm, 5 mm, 5.7 mm, and 0.2 mm, respectively. The simulated (**b**) reflection coefficients and (**c**) transmission coefficients of structure D. (**d**) Current distributions on the plane *z* = −0.635 mm of structure D at 1.57 GHz.

In consideration of practicality, all the frequencies outside the interested passband have to be rejected. First, a shunt SIR is added to the output microstrip line to acquire a new transmission zero^34^, which consists of two impedance sections, as shown in Fig. 9(a). The filter is denoted as structure D. The lengths of the two impedance sections are *l*_1_ and *l*_2_, and the widths are *w*_1_ and *w*_2_. The designing principle is to make the SIR resonance frequency fall in the vicinity of the resonant frequency for the dipole mode. Hence the waves at the dipole mode would be coupled into the SIR structure and be rejected out of the interested passband. The parameters *l*_1_, *l*_2_, *w*_1_, and *w*_2_ are optimized to 4.5 mm, 5 mm, 5.7 mm, and 0.2 mm, respectively. The reflection and transmission coefficients of the wideband filter with a shunt SIR are shown in Fig. 9(b) and (c). We can see that the dipole mode has been effectively suppressed. The current distributions on the plane *z* = −0.635 mm at 1.57 GHz (corresponding to the marked point m_1_) have been illustrated in Fig. 9(d). As expected, it can be clearly seen that the dipole mode waves have been effectively coupled into the SIR structure.

**Figure 10:**
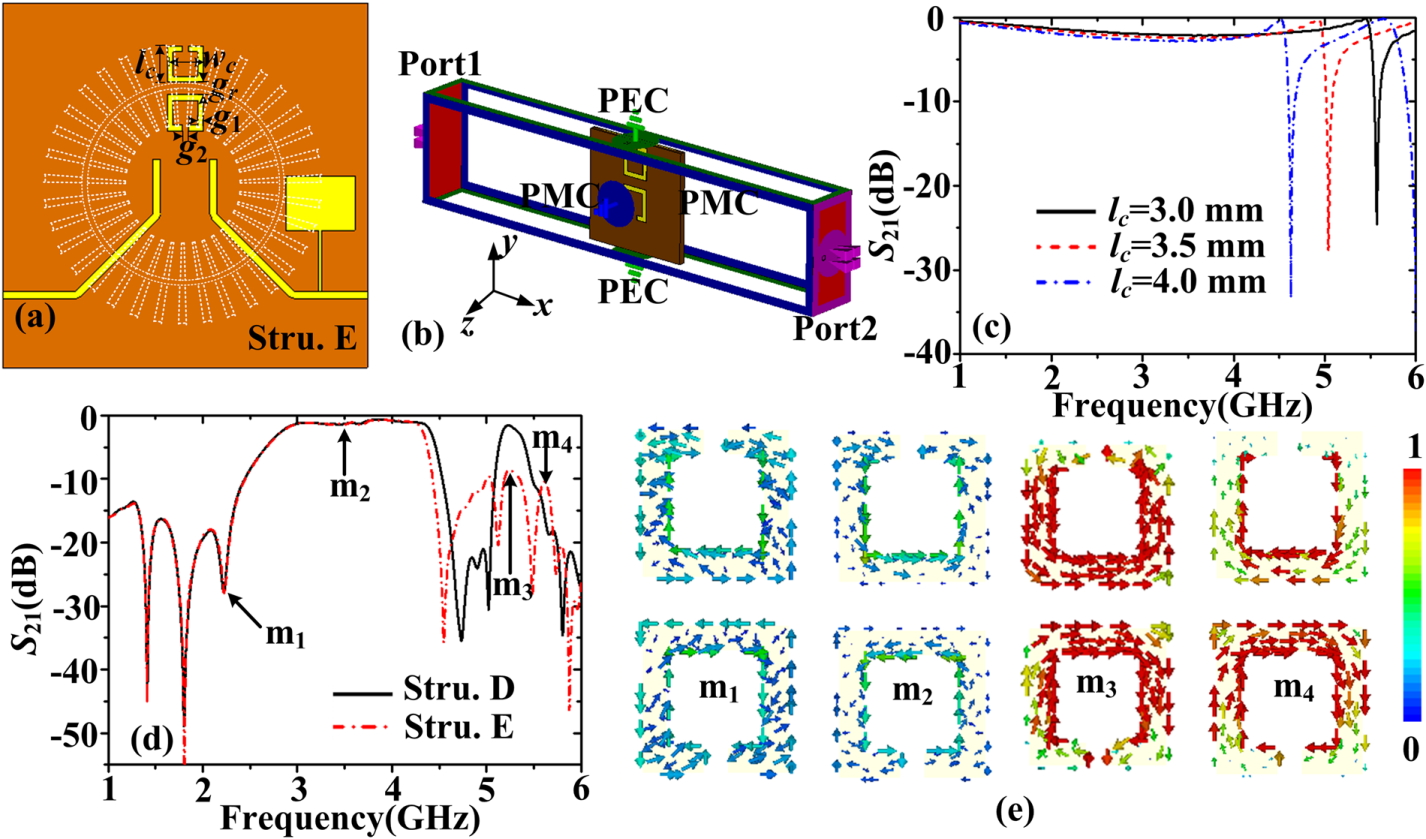
(**a**) The bottom view of the wideband plasmonic filter with a SIR and double C-shaped rings (structure E), where the values of *w*_*c*_, *g*_1_, *g*_2_, and *g*_r_ are fixed to 3 mm, 0.5 mm, 0.5 mm, and 1mm, respectively. *l*_*c*_ is optimized to 3 mm to make the octopole mode waves coupled into the double C-shaped rings. (**b**) The boundary conditions of the double C-shaped rings in the CST simulation. (**c**) The simulated transmission coefficients of the double C-shaped rings for different *l*_*c*_. (**d**) The simulated transmission coefficients of structure E. (**e**) Current distributions on the plane *z* = −0.635 mm of structure E at 2.23 GHz, 3.5 GHz, 5.24 GHz and 5.62 GHz.

Then to further reject the octopole (*n* = 4) mode, double C-shaped rings^35^ are introduced on the back of the corrugated MIM ring, as illustrated in Fig. 10(a). The structure is called as structure E. The width, length, line width and gap width of the C-shaped ring are denoted as *w*_*c*_, *l*_*c*_, *g*_1_, and *g*_2_. The values of *w*_*c*_, *g*_1_, and *g*_2_ are set as 3 mm, 0.5 mm, and 0.5 mm, respectively. The distance *g*_*r*_ between the two C-shaped rings is 1 mm. It’s known that the C-shaped ring can be efficiently excited by the transverse magnetic (TM) waves. The basic principle of rejecting the octopole mode is to make the resonant frequency of the double C-shaped rings fall in the vicinity of the resonant frequency for the octopole mode. To numerically calculate the resonant frequency of the double C-shaped rings using CST, the boundary conditions in *y* and *z* directions are set as the perfect electrical conductor (PEC) and perfect magnetic conductor (PMC) to support the TM waves, as shown in Fig. 10(b). The scattering parameters of the double C-shaped rings with the changing of *l*_*c*_ are shown in Fig. 10(c). Since the C-shaped ring can be represented by the parallel GLC model and the equivalent inductance *L* increases when *l*_*c*_ is increased, as expected the resonant frequencies red shift. Due to the strong coupling between the spoof LSPs resonator and the double C-shaped rings, it is expected that the octopole mode waves will be coupled into the double C-shaped rings and the mode can be rejected. The optimized *l*_*c*_ is 3 mm and the estimated resonant frequency is 5.57 GHz from Fig. 10(c). The transmission coefficients of the wideband filter with double C-shaped rings are shown in Fig. 10(d). It can be seen that the octopole mode has been suppressed with the transmission coefficients below −10 dB. To confirm the coupling between the spoof LSPs resonator and the double C-shaped rings, the current distributions on the C-shaped rings at 2.23 GHz, 3.5 GHz, 5.24 GHz and 5.62 GHz, which are marked as m_1_-m_4_ in Fig. 10(d), have been illustrated in Fig. 10(e). We can see that the magnitude of the current flow on the double C-shaped rings is small at m_1_-m_2_ points, thus the coupling is weak. The current on the double C-shaped rings at m_3_ and m_4_ points are big and it indicates that the coupling is strong in the vicinity of the resonant frequency of the double C-shaped rings. Hence we can validate that it is the coupling between the double C-shaped rings and the spoof LSPs resonator which brings in the rejection of the octopole mode.

## Discussion

Here we have theoretically and experimentally proposed an ultra-compact and wideband plasmonic filte based on corrugated MIM ring resonator. The proposed filter exhibits several attractive features such as compact size, wide bandwidth, low transmission loss, flat-top transmission response and sharp out-of-band suppression. Broad passband covering the quadrupole mode and the hexapole mode can be achieved by selecting proper lengths in the input and output coupling area. The mutual coupling between the input and output microstrip lines generates a new resonance mode and produces the flat-top transmission response and sharper out-of-band rejection. By introducing a shunt SIR and double C-shaped rings on the back of the substrate of the filter, the rejections of the dipole mode and the octopole mode can be achieved. The operating physical mechanism has been fully investigated, which can be expanded to THz frequencies. Simulated results agree well with the experimental results. The spoof plasmonic filter can find more applications in the highly integrated plasmonic circuits at microwave and THz frequencies.

## Methods

### Simulations and experiments

All samples are fabricated using Rogers RO3010 substrate whose relative dielectric constant is 10.2 and loss tangent is 0.0023. The numerical simulations are conducted with the help of the commercial software, CST Microwave Studio. The calculation of dispersion relations of the MIM ring resonator is based on CST eigenmode solver, where only one unit cell is analyzed and periodic boundary conditions (PBC) are used. The distributions of surface electric field and surface currents are calculated by use of CST transient solver, which is based on the finite-integral technique (FIT) method. Tetrahedral mesh and open boundary conditions are adopted. The fabricated sample is connected to Vector Network Analyzer (VNA, Agilent N5227A) to obtain the reflection and transmission coefficients.
